# Optical coherence tomography-guided vs. intravascular ultrasound-guided percutaneous coronary intervention: a systematic review and meta-analysis of randomized controlled trials

**DOI:** 10.3389/fcvm.2024.1395606

**Published:** 2024-05-31

**Authors:** Vaibhav Vats, Aarij Elahi, Sinda Hidri, Rem Ehab Abdelkader, Farhan Munaf, Jennifer Mercika Prince, Muhammad Ahsan Asif, Huzaifa Ahmad Cheema, Adeel Ahmad, Wajeeh Ur Rehman, Abdulqadir J. Nashwan, Raheel Ahmed, Vladimir Lakhter, Hafeez Ul Hassan Virk, Royce P. Vincent

**Affiliations:** ^1^ Department of Medicine, Smt. Kashibai Navale Medical College and General Hospital, Pune, India; ^2^ Department of Medicine, Royal Glamorgan Hospital, Pontyclun, United Kingdom; ^3^ Department of Internal Medicine, East Carolina University, Greenville, NC, United States; ^4^ Department of Medicine, Mansoura University, Mansoura, Egypt; ^5^ Department of Medicine, Liaquat National Medical College, Karachi, Pakistan; ^6^ Department of Haematology-Oncology, National University Hospital (NUH), Singapore, Singapore; ^7^ Department of Radiology and Medical Imaging, Jinnah Hospital, Lahore, Pakistan; ^8^ Department of Cardiology, King Edward Medical University, Lahore, Pakistan; ^9^ Department of Internal Medicine, Mass General Brigham-Salem Hospital, Salem, MA, United States; ^10^ Department of Internal Medicine, United Health Services Hospital, Johnson, NY, United States; ^11^ Hamad Medical Corporation, Doha, Qatar; ^12^ National Heart & Lung Institute, Imperial College London, London, United Kingdom; ^13^ Department of Cardiology, Royal Brompton Hospital, London, United Kingdom; ^14^ Cardiology Division, Department of Internal Medicine, Temple University Hospital, Philadelphia, PA, United States; ^15^ Department of Cardiovascular Disease, Adena Regional Medical Center, Chillicothe, OH, United States; ^16^ Department of Clinical Biochemistry, King’s College Hospital NHS Foundation Trust, London, United Kingdom; ^17^ Honorary Senior Lecturer, Faculty of Life Sciences & Medicine, King’s College London, London, United Kingdom

**Keywords:** optical coherence tomography, intravascular ultrasound, percutaneous coronary intervention, OCT, IVUS

## Abstract

**Background:**

Optical coherence tomography (OCT) and intravascular ultrasound (IVUS) are superior to coronary angiography for guiding percutaneous coronary intervention (PCI). However, whether one technique is superior to the other is inconclusive.

**Methods:**

We searched PubMed, Embase, the Cochrane Library, and ClinicalTrials.gov from inception to November 2023 for randomized controlled trials (RCTs) comparing OCT and IVUS in patients undergoing PCI. RevMan 5.4 was used to pool outcomes with risk ratio (RR) as the effect measure.

**Results:**

Six RCTs (4,402 patients) were included in this meta-analysis. There was no significant difference between the OCT- and IVUS-guided PCI groups in the risk of major adverse cardiovascular events (RR 0.87, 95% CI: 0.65, 1.16; I^2 ^= 0%) and cardiac mortality (RR 0.73, 95% CI: 0.24, 2.21; I^2 ^= 0%). The results were consistent across the subgroups of the presence or absence of left main disease (P*_interaction_* >0.1). There were no significant differences between OCT and IVUS in the risk of target lesion revascularization (RR 0.78, 95% CI: 0.47, 1.30; I^2 ^= 0%), target vessel revascularization (RR 1.06, 95% CI: 0.69, 1.62; I^2 ^= 0%), target-vessel myocardial infarction (RR 0.79, 95% CI: 0.40, 1.53; I^2 ^= 0%), stent thrombosis (RR 0.59, 95% CI: 0.12, 2.97; I^2 ^= 0%), and all-cause mortality (RR 1.01, 95% CI: 0.53, 1.90; I^2 ^= 0%).

**Conclusions:**

Our meta-analysis demonstrated similar clinical outcomes in OCT- and IVUS-guided PCI. New large-scale multicenter RCTs with long-term follow-up are required to confirm or refute our findings and provide more reliable results.

**Systematic Review Registration:**

PROSPERO, identifier, CRD42023486933

## Introduction

Despite its known limitations, coronary angiography has long been considered the gold standard for diagnosing coronary artery disease and guiding percutaneous coronary intervention (PCI) (1). More specifically, its reliance on a 2-dimensional projection falls short of fully capturing the 3-dimensional nature of the coronary lumen (2).

Recently, optical coherence tomography (OCT) and intravascular ultrasound (IVUS) have emerged as valuable tools capable of overcoming several limitations associated with coronary angiography (1). Multiple studies have indicated that IVUS- and OCT-guided PCI yield better clinical outcomes, including reduced cardiac mortality and major adverse cardiac events (MACE), compared to coronary angiography-guided PCI (3–5). IVUS optimizes and guides stent placement by providing enhanced information regarding vessel lumen dimensions, plaque characteristics, overall plaque burden, and the extent of calcification (6). OCT offers higher resolution than IVUS and can be particularly helpful in guiding PCI, especially in lipid-rich plaque and severely calcified lesions (6, 7).

While multiple trials have focused on establishing the superiority of IVUS and OCT compared to coronary angiography alone, only a limited number of studies have compared OCT directly to IVUS. Previous meta-analyses have largely focused on indirect comparisons to determine which imaging modality is superior to the other (3, 4) and, in some cases, have also included observational studies that provide a poorer quality of evidence (4, 5). Recently, the results of the largest trial to date addressing this question, the OCTIVUS trial (2,008 patients), have been published (8). Therefore, we sought to conduct this meta-analysis to compare the outcomes of OCT-guided PCI to IVUS-guided PCI using data from randomized controlled trials (RCTs).

## Methods

This systematic review and meta-analysis was performed according to the Preferred Reporting Items for Systematic Reviews and Meta-analyses (PRISMA) statement and the Cochrane Collaboration guidelines (9, 10). The protocol has been registered with the International Prospective Register of Systematic Reviews (PROSPERO) under the following identifier (CRD42023486933). No form of ethical approval was required for our study as only publicly available data was used.

### Data sources and searches

The following databases were searched from inception to November 2023: MEDLINE (via PubMed), Embase, the Cochrane Central Register of Controlled Trials (CENTRAL, via the Cochrane Library), and ClinicalTrials.gov. A search strategy comprising relevant medical subject headings (MeSH) and keywords was utilized and has been reported in detail in Supplementary Table S1. In addition, a partial grey literature search (via Google Scholar) and backward citation tracking using relevant medical literature were also conducted.

### Eligibility criteria

Studies meeting the following criteria were included: (1) study design: RCTs only; (2) population: patients undergoing PCI regardless of indication; (3) intervention: OCT-guided PCI; (4) comparator: IVUS-guided PCI; (5) outcomes: reporting of any outcome of interest. For multi-arm trials, only data for the IVUS and OCT arms were obtained.

The exclusion criteria included the following: (1) all study designs other than RCTs, such as quasi-randomized trials and observational studies; (2) studies conducted on animals; and (3) single-arm trials.

### Study selection and data extraction

All literature retrieved from our search was imported into Mendeley Desktop 1.19.8, where all duplicates were removed; studies were then transferred to Rayyan to begin the screening process. Two reviewers independently screened the title and abstract of all relevant papers, followed by a full-text screening. The two authors resorted to discussion and consultation with a third author to resolve conflicts.

Data regarding study characteristics (including authors, trial name, and study location), patient population (including age and gender), cardiac disease (acute coronary syndrome, left main disease, multi-vessel disease, as well as lesion type and type of stent), study follow-up, and primary and secondary outcomes were extracted into a pre-piloted Excel spreadsheet.

### Outcomes

Our primary outcomes were the incidence of MACE and cardiac mortality. Our secondary outcomes included target lesion revascularization (TLR), target vessel revascularization (TVR), target vessel myocardial infarction (MI), stent thrombosis, and all-cause mortality.

### Risk of bias assessment

In order to assess the internal validity of the included RCTs, two authors independently applied the revised Cochrane “Risk of Bias” tool (RoB 2.0) (11). RoB 2.0 assesses the risk of bias using the following five domains: randomization process, deviations from intended interventions, missing outcome data, measurement of outcome, and selective outcome reporting. The studies were assigned a rating of low risk of bias, some concerns, or a high risk of bias. Any disagreement was resolved by consulting a third reviewer.

### Data synthesis

The meta-analysis was carried out using Review Manager (RevMan, Version 5.4; The Cochrane Collaboration, Copenhagen, Denmark) under a random-effects model. Risk ratio (RR) with the corresponding 95% confidence interval (CI) was utilized as the effect measure. We used the I^2^ and Chi^2^ statistics to report statistical heterogeneity (I^2^ = 25%–50% was considered mild, 50%–75% moderate, and >75% severe heterogeneity). Additionally, a subgroup analysis based on including or excluding patients with left main disease in the studies was undertaken for our primary outcomes. A *P*-value of <0.1 was considered critical for the test for subgroup differences (12). Furthermore, a sensitivity analysis was conducted by excluding studies at a high risk of bias. It is not recommended to assess publication bias when the number of included studies is less than 10; nevertheless, for our primary outcomes, we constructed funnel plots and ran Egger's regression test to evaluate for publication bias.

## Results

### Study selection and characteristics

**Figure 1:**
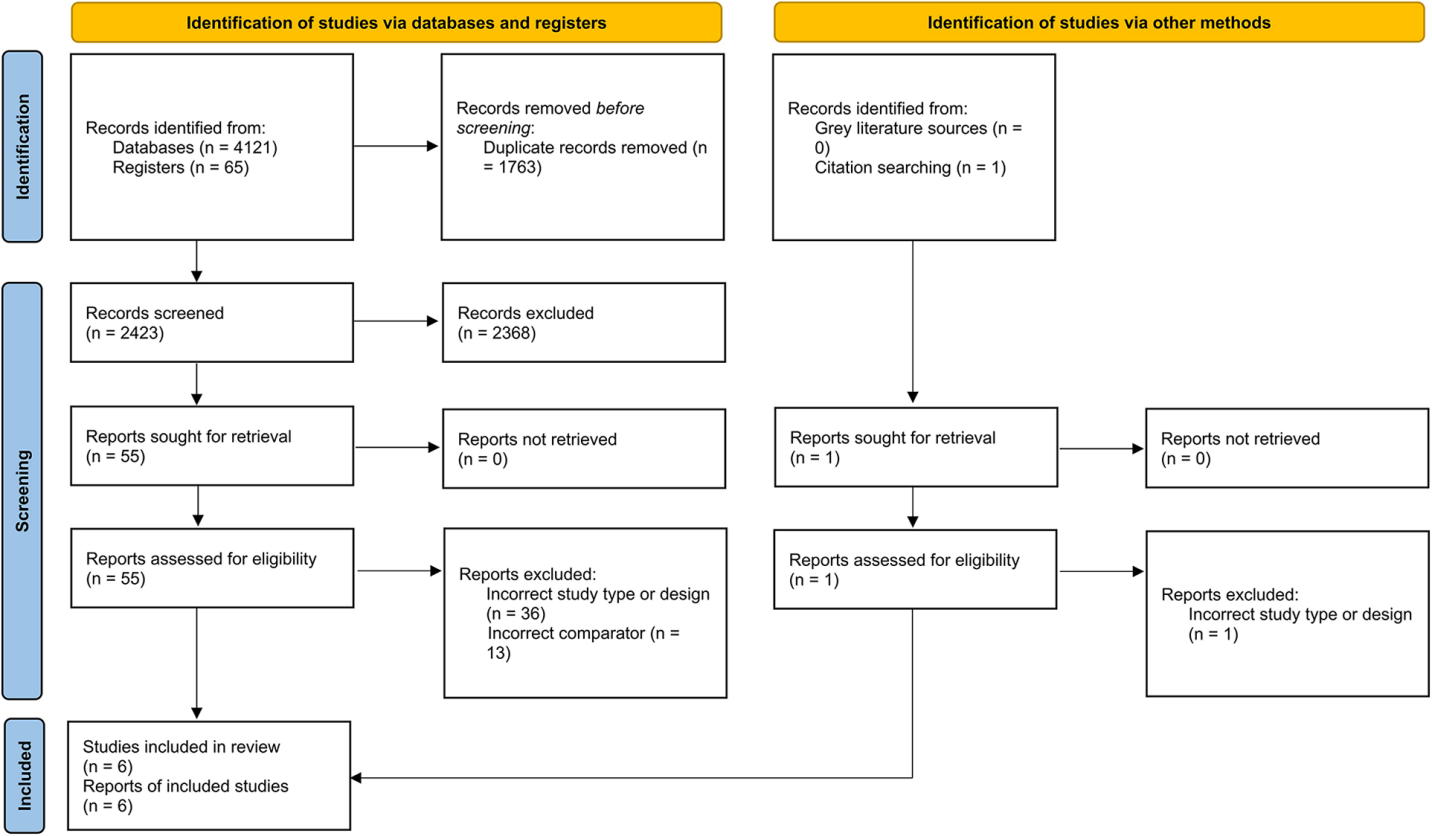
PRISMA 2020 flowchart of the study selection procedure.

A total of 6 RCTs (4,402 patients) were included in this meta-analysis after a thorough systemic search (Figure 1) (13–17). Two of these studies were from Japan (13, 16), two from South Korea (8, 14), and one from Brazil (15); the remaining study was conducted in 8 countries (17). Three RCTs included patients with left main disease (8, 14, 16). The types of lesions differed between the trials, including thrombotic lesions, calcifications, and bifurcation lesions. Detailed information about each study is provided in Tables 1, 2.

**Table 1:** Characteristics of included studies and main baseline clinical characteristics of included patients.

Study ID	Trial name	Location	Study arms	No. of patients	Age (years)	Male (%)	ACS (%)	LM (%)	Diabetes (%)	Previous MI (%)	Previous PCI (%)	Lesion type	Stent type	MACE definition	Follow-up duration
Kubo et al. (16)	OPINION	Japan	OCT vs. IVUS	829 (414 vs. 415)	69 ± 9 vs. 68 ± 9	76.5 vs. 79.5	11.7 vs. 13.1	74.5 vs. 70.1	41 vs. 40.7	17 vs. 15.1	34 vs. 34.6	Thrombus, bifurcation, moderate or heavy calcification, long lesions	Biolimus-eluting stent	Composite of cardiac death, MI, or TLR	12 months
Muramatsu et al. (13)	MISTIC-1	Japan	OCT vs. IVUS	109 (54 vs. 55)	72 vs. 71	75.9 vs. 80.0	NR	0 vs. 0	50 vs. 43.6	35.2 vs. 29.1	44.4 vs. 47.3	Thrombotic lesions, Moderate or severe calcification	Biolimus A9-eluting metallic stents	Composite of cardiac death, target-vessel MI, or TLR	36 months
Ali et al. (17)	ILUMIEN III	Multicenter (29 hospitals in 8 countries)	OCT vs. IVUS vs. angiography^a^	450 (158 vs. 146 vs. 146)	66 (59–72) vs. 66 (61–72)	69 vs. 73	33 vs. 36	0 vs. 0	33 vs. 36	NR	NR	Thrombotic lesions, calcified lesions (moderate to severe)	Everolimus-eluting, zotarolimus-eluting, sirolimus-eluting, or biolimus-eluting stents	Composite of death, MI, stent thrombosis, or repeat revascularization.	12 months
Chamié et al. (15)	iSIGHT	Brazil	OCT vs. IVUS vs. angiography^a^	151 (51 vs. 51 vs. 49)	59.9 ± 8.9 vs. 59.3 ± 10.4	60.8 vs. 72.0	Unstable angina/NSTEMI: 39.2 vs. 44.0 Recent MI: 17.7 vs. 20.0	0 vs. 0	33.3 vs. 40	29.4 vs. 34	23.5 vs. 26	≥1 target lesion in ≥1 native coronary with a reference diameter ranging from 2.25 to 4.0 mm. Significant (≥50%) stenosis in the left main stem, aorto-ostial lesions, chronic total occlusions, and bifurcation lesions were excluded.	*Resolute, Xience, Promus Element, Biomatrix*	Composite of cardiac death, MI and TLR	30 months
Lee et al. (14)	RENOVATE-COMPLEX-PCI	South Korea	OCT or IVUS vs. angiography	1,639 (1,092 vs. 547)	65.3 ± 10.3 vs. 66.0 ± 10.0	79.6 vs. 78.8	51.3 vs. 49.7	12.6 vs. 9.9	36.1 vs. 40.8	6.9 vs. 7.7	24.1 vs. 24.5	Complex coronary-artery lesions	Biodegradable or biocompatible polymer-coated everolimus-eluting stents	Composite of cardiac death, target-vessel MI, or TVR.	36 months
Kang et al. (8)	OCTIVUS	South Korea	OCT vs. IVUS	2,008 (1,005 vs. 1,003)	64.3 + 10.3 vs. 65.1 + 10.5	78.6 vs. 78.3	23.5 vs. 23.3	11.5 vs. 14.8	32.3 vs. 34.4	7.8 vs. 6.3	22.5 vs. 20.1	Complex coronary-artery lesions	Contemporary drug-eluting stents	Composite of cardiac death, target-vessel MI, or TLR	12 months

ACS, acute coronary syndrome; LM, left main disease; MACE, major adverse cardiovascular events; OCT, optical coherence tomography; IVUS, intravascular ultrasound; NR, not reported; MI, myocardial infarction; TLR, target lesion revascularization; TVR, target vessel revascularization.

^a^ Data from the angiography arm was excluded.

**Table 2:** Angiographic and procedural characteristics of included trials.

Study ID	Trial name	Study arms	Stent diameter (mm)	Stent length (mm)	Max balloon diameter (mm)	Lesion length (mm)	Multi-vessel disease (%)	LAD (*n*)	LCX (*n*)	RCA (*n*)
Kubo et al. (16)	OPINION	OCT vs. IVUS	2.92 vs. 2.99	25.9 vs. 24.8	3.1 vs. 3.3	17.73 vs. 17.56	NR	223 vs. 197	84 vs. 87	102 vs. 117
Muramatsu et al. (13)	MISTIC-1	OCT vs. IVUS	3.00 vs. 3.00	18.0 vs. 18.0	3.25 vs. 3.50	11.7 vs. 11.1	37.1 vs. 43.6	31 vs. 25	13 vs. 17	18 vs. 22
Ali et al. (17)	ILUMIEN III	OCT vs. IVUS vs. angiography^a^	3.00 vs. 3.13	23 vs. 24	3.5 vs. 3.5	15.3 vs. 15.3	NR	52 vs. 50	27 vs. 27	22 vs. 22
Chamié et al. (15)	iSIGHT	OCT vs. IVUS vs. angiography^a^	3.26 vs. 3.31	28.57 vs. 32.51	3.5 vs. 3.5	23.61 vs. 21.10	NR	19 vs. 22	12 vs. 10	20 vs. 19
Lee et al. (14)	RENOVATE-COMPLEX-PCI	OCT or IVUS vs. angiography	3.1 vs. 3.0	38.0 vs. 36.9	3.5 vs. 3.5	28.4 vs. 26.8	68.7 vs. 66.3	701 vs. 376	313 vs. 151	445 vs. 215
Kang et al. (8)	OCTIVUS	OCT vs. IVUS	3.27 vs. 3.37	47.2 vs. 47.8	3.64 vs. 3.78	29.9 vs. 29.3	60.5 vs. 62.7	656 vs. 640	210 vs. 204	290 vs. 294

OCT, optical coherence tomography; IVUS, intravascular ultrasound.

^a^ Data from the angiography arm was excluded.

### Risk of bias assessment

**Figure 2:**
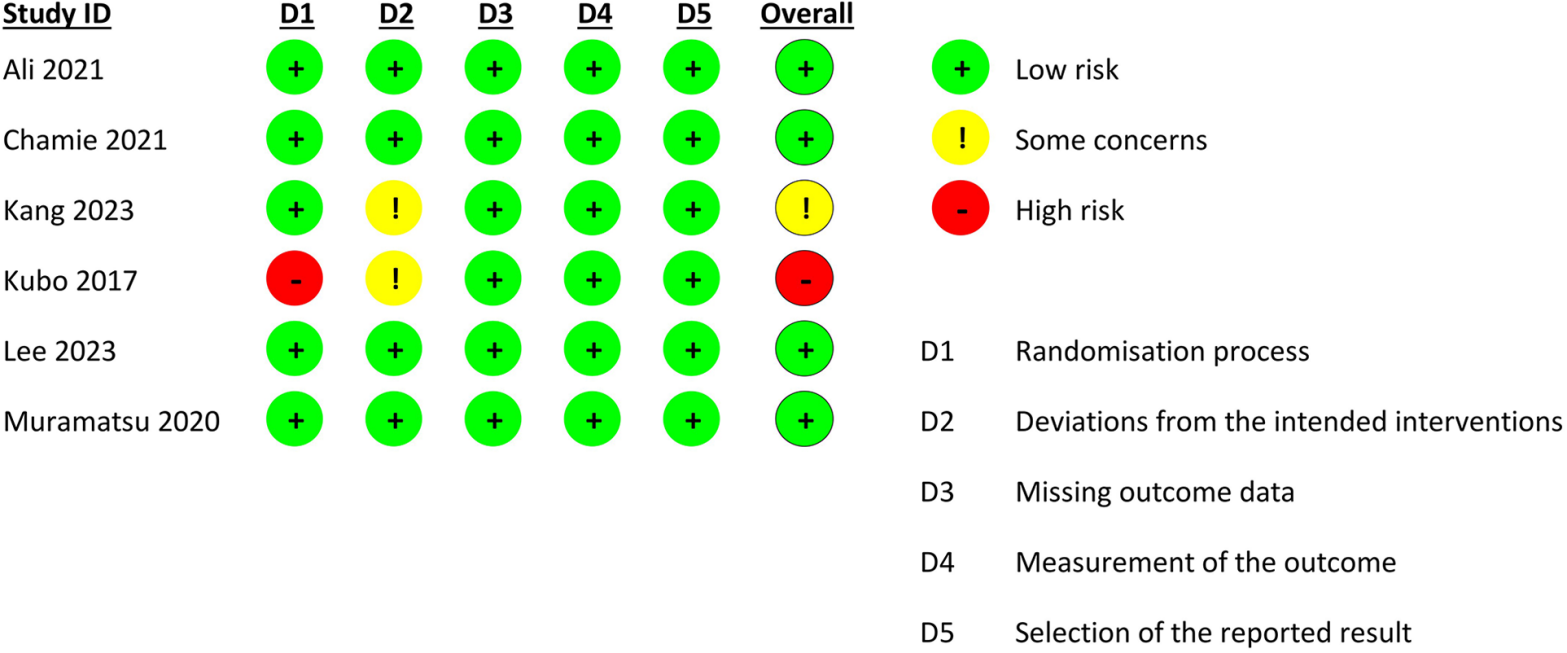
Quality assessment of included trials.

Four studies were deemed to be at a low risk of bias (13–15, 17), one study had some concerns due to deviations from intended interventions (8), and one study had a high risk of bias due to issues in the domain of randomization (Figure 2) (16).

### Results of the meta-analysis

#### Primary outcomes

**Figure 3:**
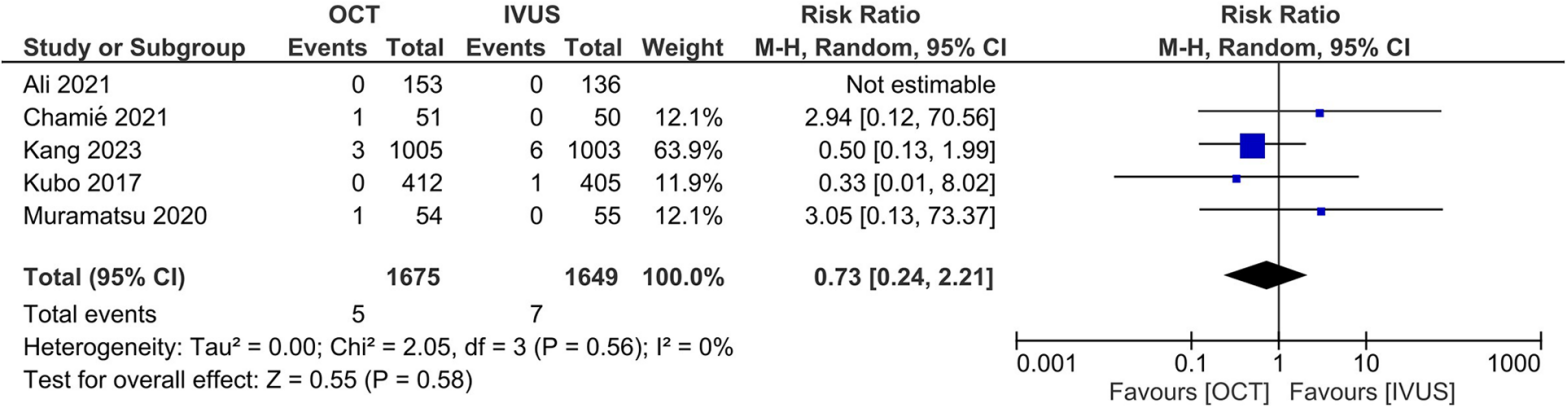
Effect of OCT- vs. IVUS-guided PCI on major adverse cardiovascular events (MACE).

**Figure 4:**
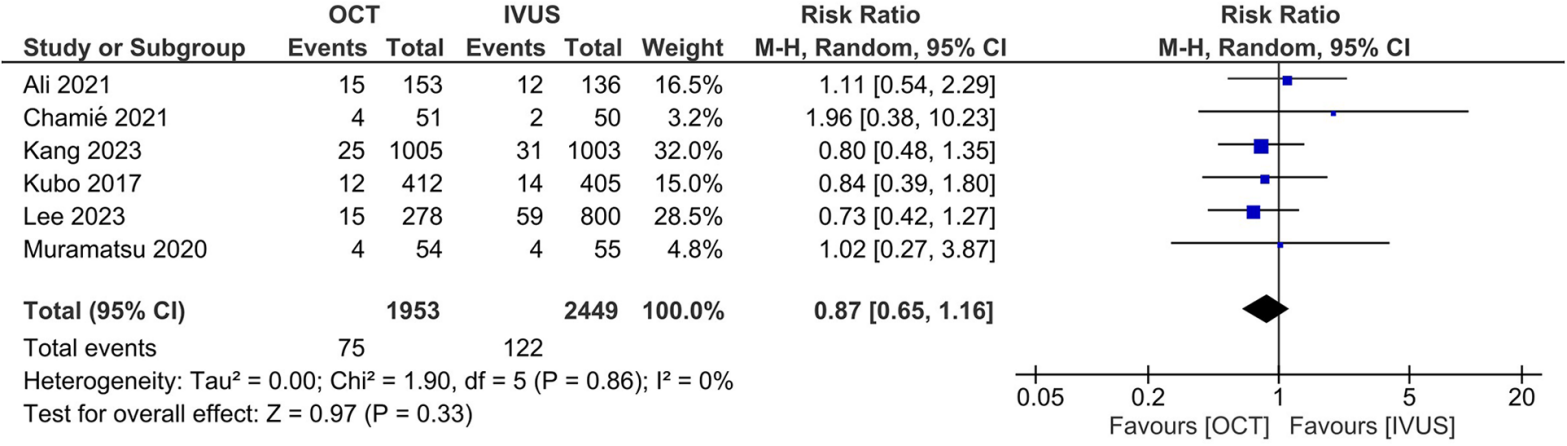
Effect of OCT- vs. IVUS-guided PCI on cardiac mortality.

There was no significant difference between the OCT- and IVUS-guided PCI groups in the risk of MACE (RR 0.87, 95% CI: 0.65, 1.16; I^2 ^= 0%; Figure 3) and cardiac mortality (RR 0.73, 95% CI: 0.24, 2.21; I^2 ^= 0%; Figure 4). The results were consistent across the subgroups of the presence or absence of left main disease (P*_interaction_* >0.1; Supplementary Figures S1, S2). A sensitivity analysis excluding the trial with a high risk of bias demonstrated similar findings. There was no indication of publication bias in either of the two primary outcomes (Egger's *P*-value >0.05; Supplementary Figures S3, S4).

#### Secondary outcomes

There were no significant differences between OCT and IVUS in the risk of TLR (RR 0.78, 95% CI: 0.47, 1.30; I^2 ^= 0%; Supplementary Figure S5), TVR (RR 1.06, 95% CI: 0.69, 1.62; I^2 ^= 0%; Supplementary Figure S6), target-vessel MI (RR 0.79, 95% CI: 0.40, 1.53; I^2 ^= 0%; Supplementary Figure S7), stent thrombosis (RR 0.59, 95% CI: 0.12, 2.97; I^2 ^= 0%; Supplementary Figure S8), and all-cause mortality (RR 1.01, 95% CI: 0.53, 1.90; I^2 ^= 0%; Supplementary Figure S9). The results did not change substantially upon exclusion of the trial with a high risk of bias.

## Discussion

To the best of our knowledge, this is the most comprehensive meta-analysis on this topic to date. Our analysis comparing OCT with IVUS guidance demonstrated no difference between the two imaging modalities regarding the risk of MACE, cardiac mortality, TLR, TVR, target-vessel MI, stent thrombosis, and all-cause mortality. The results were consistent regardless of the presence or absence of left main disease in the pooled patient analysis.

These findings align with previous analyses comparing the same outcomes between the two modalities (3–5, 18), although there has been some indication that IVUS might be the better imaging modality (3). Nevertheless, the prior meta-analyses suffered from many limitations, including indirect comparisons, the incorporation of observational studies (which confer the risk of confounding bias), and the inclusion of only a few small RCTs, which provided low statistical power. Our analysis focused only on randomized trials that directly compared OCT and IVUS and had increased power due to the inclusion of recent large-scale RCTs, therefore providing more reliable results.

The finer resolution and image quality of both OCT and IVUS allow for a better understanding of luminal anatomy, plaque location, and precise vessel dimensions, which allow for improved stent sizing and positioning (19, 20). Their improved ability to discern stent malpositioning, under-expansion, and edge dissection elucidates the improved clinical outcomes compared to conventional angiographic guidance (3, 4). However, when compared to each other, our findings show no evidence of superior clinical benefit of either OCT or IVUS. These results further consolidate the guidelines of the American Heart Association/American College of Cardiology/Society of Cardiovascular Angiography & Interventions, which state that OCT and IVUS are justifiable alternatives to each other, with the sole exception of ostial left main disease, in which case IVUS is preferred (21). Nevertheless, it is important to note that due to the low incidence of some outcomes, future large trials and subsequent meta-analyses will be needed to attain adequate statistical power to elucidate whether either of these two techniques is superior.

The majority of studies indicate that OCT guidance at the time of PCI leads to the use of larger stent diameters than would have been chosen based on angiography alone. However, when compared to IVUS, OCT has been shown to result in a smaller minimal stent area (MSA) (22, 23). Although the use of infrared light-based technology behind OCT allows the production of detailed cross-sectional imaging of the luminal wall with a 10-fold higher resolution compared to IVUS, its relative inability to traverse through the entire vessel wall limits the complete assessment of the full vessel dimension (24, 25). The ultrasound-guided approach in IVUS allows for deeper transmittance along with much better and consistent visualization of the external elastic lamina, elucidating the entire vessel wall thickness (5). Nevertheless, these differences between the two techniques did not translate to any differences in relevant clinical outcomes in our analysis.

The repeated need to clear the blood columns by saline or contrast to generate precise imaging in OCT-guided PCI adds to its procedural complexity, questioning its application in contrast-sensitive patients with compromised renal function and potentially limiting its widespread use (26). A recent report showed that the application of OCT and IVUS guidance is limited to only 0.6% and 8.7% of PCIs for MI in the US, respectively (26); factors restricting their extensive use include limited operator expertise, higher financial burden, and the lack of necessary technology in some hospitals (27, 28).

There are some limitations to our meta-analysis. Although all of our outcomes had low statistical heterogeneity, some residual heterogeneity likely exists due to differences in anatomical and procedural characteristics between the trials. Additionally, since we did not have access to individual patient data, we could not extensively investigate potential effect modifiers in our study-level analysis. Furthermore, despite our meta-analysis being the largest one to date, it may still be underpowered for some outcomes. Lastly, the impact of OCT vs. IVUS on long-term outcomes is uncertain due to a lack of longer follow-ups; further large-scale RCTs with more extensive follow-ups are required to confirm our findings and provide conclusive proof.

## Conclusions

Our meta-analysis comparing OCT-guided PCI with IVUS-guided PCI demonstrated no significant difference between the two modalities regarding the incidence of MACE, cardiac death, TLR, TVR, target-vessel MI, stent thrombosis, and all-cause mortality. The choice of the imaging modality will depend on the availability of necessary technology and resources, and operator expertise. New large-scale multicenter RCTs with long-term follow-up are required to confirm or refute our findings and provide more reliable results.

## Data Availability

The raw data supporting the conclusions of this article will be made available by the authors, without undue reservation.
