# Supplementary material for: Optical coherence tomography-guided vs. intravascular ultrasound-guided percutaneous coronary intervention: a systematic review and meta-analysis of randomized controlled trials
Source: Front Cardiovasc Med. 2024 May 31;11:1395606. doi: 10.3389/fcvm.2024.1395606 (PMC11176458; doi:10.3389/fcvm.2024.1395606)

Supplementary Table 1. Search strategy for databases.

**PubMed**	((intravascular ultrasound[Title/Abstract]) OR (IVUS[Title/Abstract])) AND ((optical coherence tomography[Title/Abstract]) OR (optical frequency domain[Title/Abstract]) OR (OCT[Title/Abstract])) AND ((percutaneous coronary intervention[Title/Abstract]) OR (coronary intervention[Title/Abstract]) OR (stent implantation[Title/Abstract]) OR (angioplasty[Title/Abstract]))
**Embase**	('intravascular ultrasound':ab,ti OR ivus:ab,ti) AND ('optical coherence tomography':ab,ti OR 'optical frequency domain':ab,ti OR oct:ab,ti) AND ('percutaneous coronary intervention':ab,ti OR 'coronary intervention':ab,ti OR 'stent implantation':ab,ti OR angioplasty:ab,ti)

Supplementary Figure 1. Subgroup analysis of the effect of OCT vs. IVUS on the risk of major adverse cardiovascular events.

	OCT		IVUS			Risk Ratio
Study or Subgroup	Events	Total	Events	Total	Weight	M-H, Random, 95% CI
**1.8.1 Presence of LM disease**						
Kang 2023	25	1005	31	1003	32.0%	0.80 [0.48, 1.35]
Kubo 2017	12	412	14	405	15.0%	0.84 [0.39, 1.80]
Lee 2023	15	278	59	800	28.5%	0.73 [0.42, 1.27]
**Subtotal (95% CI)**		**1695**		**2208**	**75.5%**	**0.78 [0.56, 1.10]**
Total events	52		104			
Heterogeneity: Tau² = 0.00; Chi² = 0.11, df = 2 (P = 0.95); I² = 0%						
Test for overall effect: Z = 1.41 (P = 0.16)						
**1.8.2 Absence of LM disease**						
Ali 2021	15	153	12	136	16.5%	1.11 [0.54, 2.29]
Chamié 2021	4	51	2	50	3.2%	1.96 [0.38, 10.23]
Muramatsu 2020	4	54	4	55	4.8%	1.02 [0.27, 3.87]
**Subtotal (95% CI)**		**258**		**241**	**24.5%**	**1.18 [0.65, 2.13]**
Total events	23		18			
Heterogeneity: Tau² = 0.00; Chi² = 0.44, df = 2 (P = 0.80); I² = 0%						
Test for overall effect: Z = 0.53 (P = 0.59)						
**Total (95% CI)**		**1953**		**2449**	**100.0%**	**0.87 [0.65, 1.16]**
Total events	75		122			
Heterogeneity: Tau² = 0.00; Chi² = 1.90, df = 5 (P = 0.86); I² = 0%						
Test for overall effect: Z = 0.97 (P = 0.33)						
Test for subgroup differences: Chi² = 1.35, df = 1 (P = 0.24), I² = 26.2%						

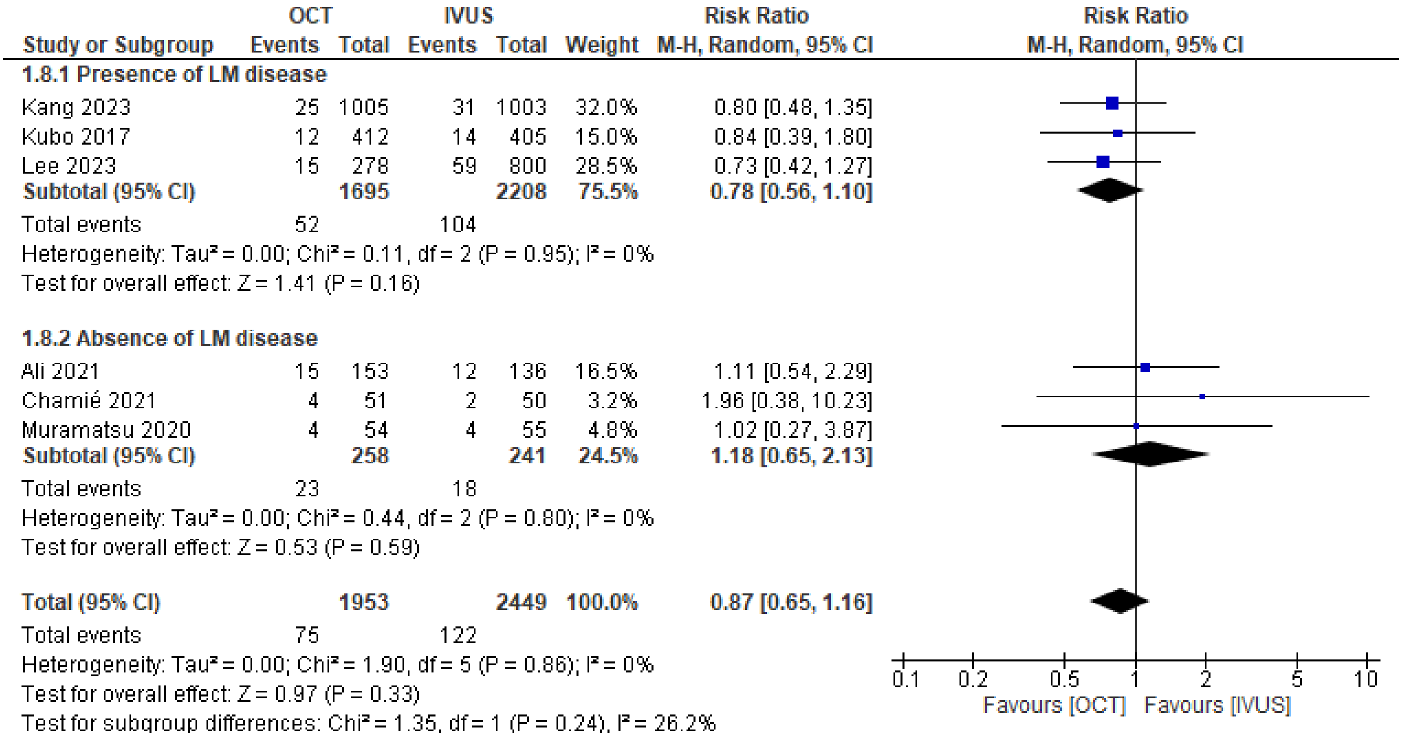

Supplementary Figure 2. Subgroup analysis of the effect of OCT vs. IVUS on the risk of cardiac mortality.

Study or Subgroup	OCT Events	OCT Total	IVUS Events	IVUS Total	Weight	Risk Ratio M-H, Random, 95% CI
**1.9.1 Presence of LM disease**						
Kang 2023	3	1005	6	1003	63.9%	0.50 [0.13, 1.99]
Kubo 2017	0	412	1	405	11.9%	0.33 [0.01, 8.02]
**Subtotal (95% CI)**		**1417**		**1408**	**75.8%**	**0.47 [0.13, 1.66]**
Total events	3		7			
Heterogeneity: Tau² = 0.00; Chi² = 0.06, df = 1 (P = 0.81); I² = 0%						
Test for overall effect: Z = 1.18 (P = 0.24)						
**1.9.2 Absence of LM disease**						
Ali 2021	0	153	0	136		Not estimable
Chamié 2021	1	51	0	50	12.1%	2.94 [0.12, 70.56]
Muramatsu 2020	1	54	0	55	12.1%	3.05 [0.13, 73.37]
**Subtotal (95% CI)**		**258**		**241**	**24.2%**	**3.00 [0.32, 28.36]**
Total events	2		0			
Heterogeneity: Tau² = 0.00; Chi² = 0.00, df = 1 (P = 0.99); I² = 0%						
Test for overall effect: Z = 0.96 (P = 0.34)						
**Total (95% CI)**		**1675**		**1649**	**100.0%**	**0.73 [0.24, 2.21]**
Total events	5		7			
Heterogeneity: Tau² = 0.00; Chi² = 2.05, df = 3 (P = 0.56); I² = 0%						
Test for overall effect: Z = 0.55 (P = 0.58)						
Test for subgroup differences: Chi² = 1.99, df = 1 (P = 0.16), I² = 49.8%						

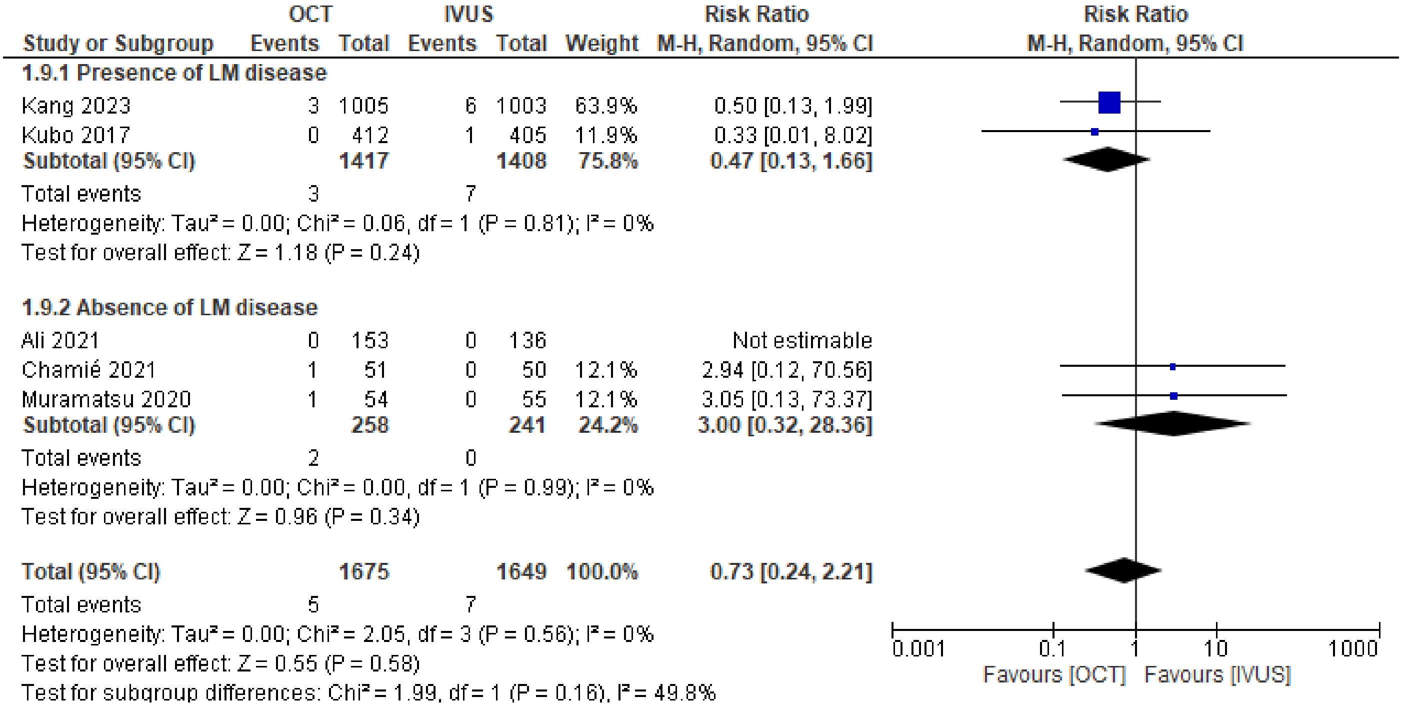

Supplementary Figure 3. Funnel plot and Egger's test for major adverse cardiovascular events.

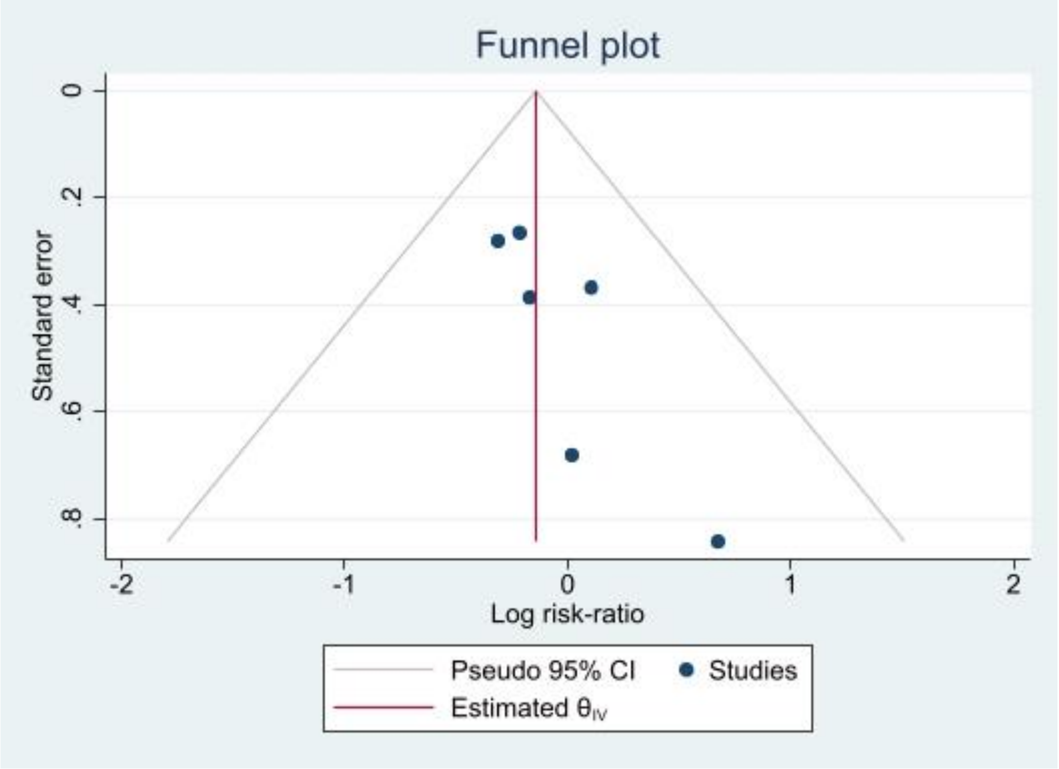


```
Regression-based Egger test for small-study effects
Random-effects model
Method: DerSimonian-Laird

H0: beta1 = 0; no small-study effects
            beta1 =       1.26
      SE of beta1 =      1.154
                z =       1.09
       Prob > |z| =     0.2745
```

Supplementary Figure 4. Funnel plot and Egger's test for cardiac mortality.

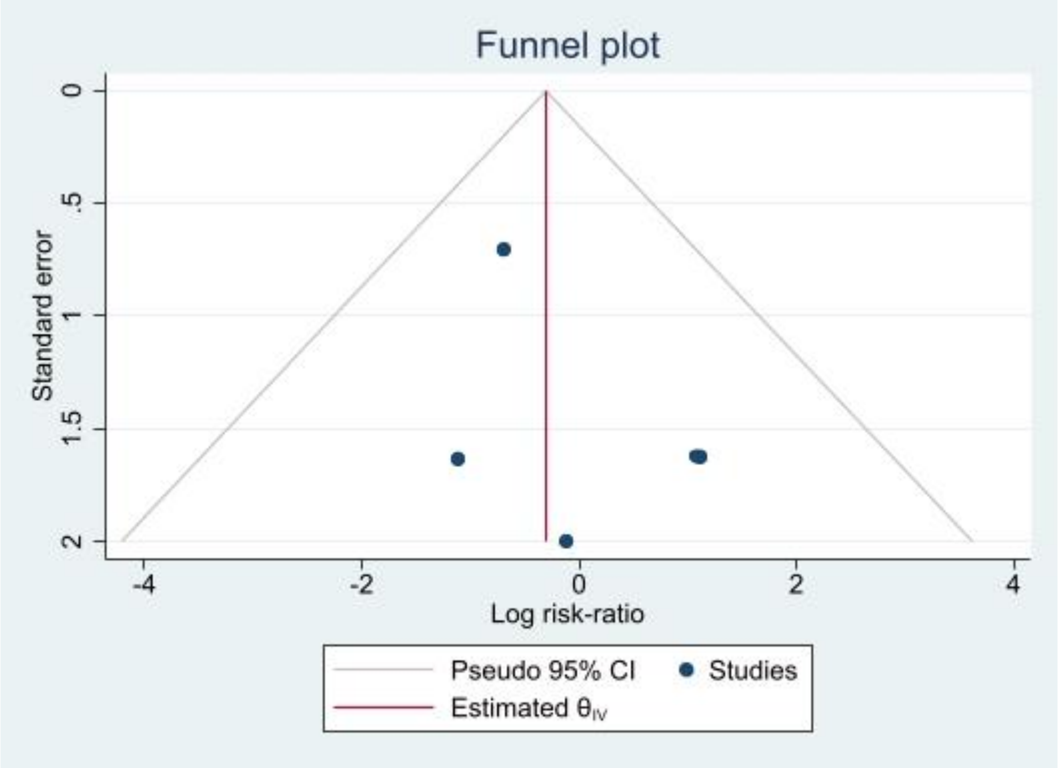


```
Regression-based Egger test for small-study effects
Random-effects model
Method: DerSimonian-Laird

H0: beta1 = 0; no small-study effects
            beta1 =       0.90
      SE of beta1 =      1.100
                z =       0.82
       Prob > |z| =     0.4122
```

Supplementary Figure 5. OCT vs. IVUS on the risk of target lesion revascularization.

	OCT		IVUS			Risk Ratio
Study or Subgroup	Events	Total	Events	Total	Weight	M-H, Random, 95% CI
Ali 2021	2	153	4	136	9.3%	0.44 [0.08, 2.39]
Chamié 2021	1	51	0	50	2.6%	2.94 [0.12, 70.56]
Kang 2023	11	1005	14	1003	42.6%	0.78 [0.36, 1.72]
Kubo 2017	11	412	12	405	40.3%	0.90 [0.40, 2.02]
Muramatsu 2020	1	54	3	55	5.3%	0.34 [0.04, 3.16]
**Total (95% CI)**		**1675**		**1649**	**100.0%**	**0.78 [0.47, 1.30]**
Total events	26		33			

Heterogeneity: Tau² = 0.00; Chi² = 1.76, df = 4 (P = 0.78); I² = 0%

Test for overall effect: Z = 0.96 (P = 0.34)

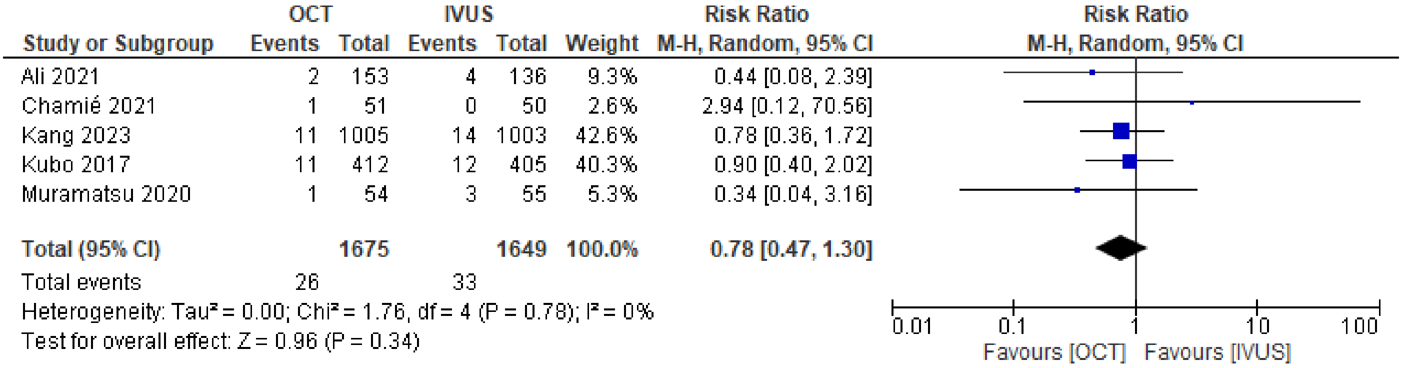

Supplementary Figure 6. OCT vs. IVUS on the risk of target vessel revascularization.

	OCT		IVUS			Risk Ratio
Study or Subgroup	Events	Total	Events	Total	Weight	M-H, Random, 95% CI
Ali 2021	4	153	3	136	8.2%	1.19 [0.27, 5.20]
Kang 2023	14	1005	16	1003	35.5%	0.87 [0.43, 1.78]
Kubo 2017	20	412	17	405	45.0%	1.16 [0.61, 2.18]
Muramatsu 2020	5	54	4	55	11.3%	1.27 [0.36, 4.49]
**Total (95% CI)**		**1624**		**1599**	**100.0%**	**1.06 [0.69, 1.62]**
Total events	43		40			

Heterogeneity: Tau² = 0.00; Chi² = 0.46, df = 3 (P = 0.93); I² = 0%

Test for overall effect: Z = 0.27 (P = 0.79)

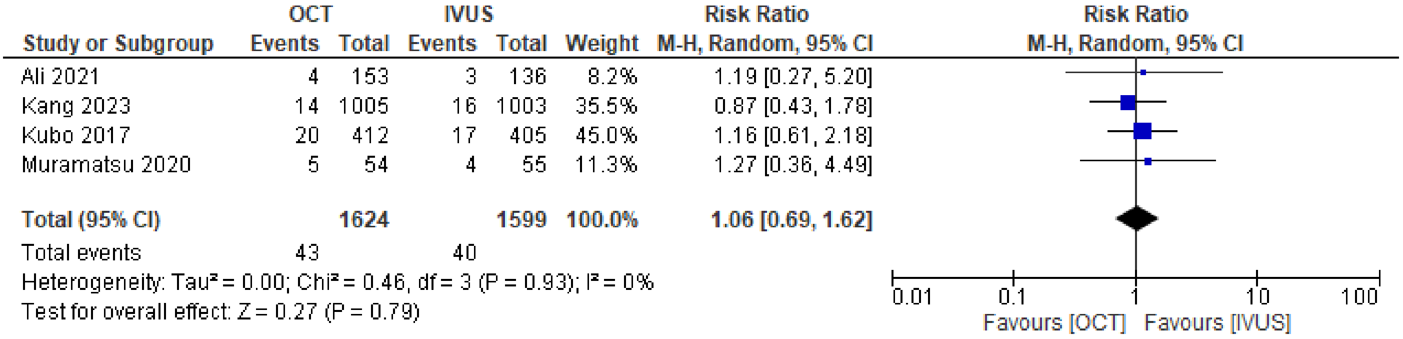

Supplementary Figure 7. OCT vs. IVUS on the risk of target vessel myocardial infarction.

	OCT		IVUS			Risk Ratio
**Study or Subgroup**	**Events**	**Total**	**Events**	**Total**	**Weight**	**M-H, Random, 95% CI**
Ali 2021	1	153	1	136	5.8%	0.89 [0.06, 14.07]
Chamié 2021	2	51	1	50	7.9%	1.96 [0.18, 20.94]
Kang 2023	9	1005	14	1003	64.3%	0.64 [0.28, 1.48]
Kubo 2017	2	412	3	405	14.0%	0.66 [0.11, 3.90]
Muramatsu 2020	2	54	1	55	7.9%	2.04 [0.19, 21.81]
**Total (95% CI)**		**1675**		**1649**	**100.0%**	**0.79 [0.40, 1.53]**
Total events	16		20			

Heterogeneity: Tau² = 0.00; Chi² = 1.47, df = 4 (P = 0.83); I² = 0%
Test for overall effect: Z = 0.71 (P = 0.48)

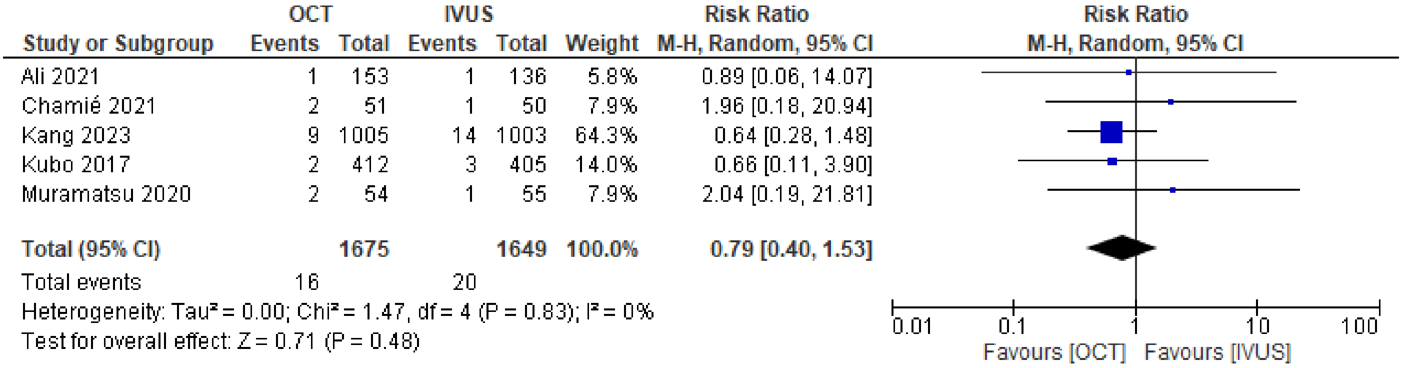

Supplementary Figure 8. OCT vs. IVUS on the risk of stent thrombosis.

	OCT		IVUS			Risk Ratio
**Study or Subgroup**	**Events**	**Total**	**Events**	**Total**	**Weight**	**M-H, Random, 95% CI**
Ali 2021	1	153	0	136	25.8%	2.67 [0.11, 64.97]
Chamié 2021	0	51	0	50		Not estimable
Kang 2023	0	1005	2	1003	28.5%	0.20 [0.01, 4.15]
Kubo 2017	1	412	2	405	45.7%	0.49 [0.04, 5.40]
Muramatsu 2020	0	54	0	55		Not estimable
**Total (95% CI)**		**1675**		**1649**	**100.0%**	**0.59 [0.12, 2.97]**
Total events	2		4			

Heterogeneity: Tau² = 0.00; Chi² = 1.37, df = 2 (P = 0.50); I² = 0%
Test for overall effect: Z = 0.64 (P = 0.52)

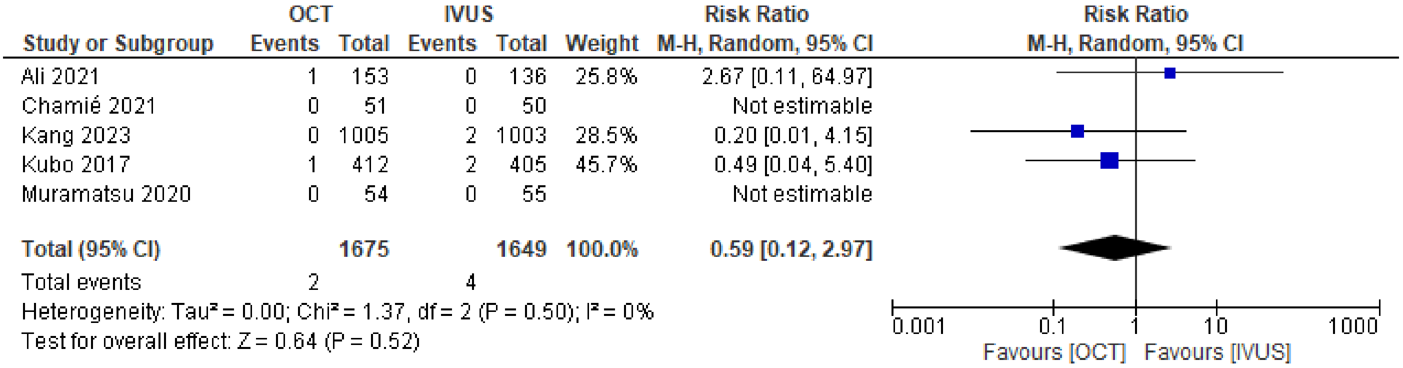

Supplementary Figure 9. OCT vs. IVUS on the risk of all-cause mortality.

	OCT		IVUS			Risk Ratio
Study or Subgroup	Events	Total	Events	Total	Weight	M-H, Random, 95% CI
Ali 2021	0	153	0	136		Not estimable
Chamié 2021	2	51	0	50	4.5%	4.90 [0.24, 99.66]
Kang 2023	10	1005	14	1003	62.1%	0.71 [0.32, 1.60]
Kubo 2017	3	412	1	405	7.9%	2.95 [0.31, 28.23]
Muramatsu 2020	5	54	4	55	25.5%	1.27 [0.36, 4.49]
**Total (95% CI)**		**1675**		**1649**	**100.0%**	**1.01 [0.53, 1.90]**
Total events	20		19			

Heterogeneity: Tau² = 0.00; Chi² = 2.78, df = 3 (P = 0.43); I² = 0%

Test for overall effect: Z = 0.02 (P = 0.98)

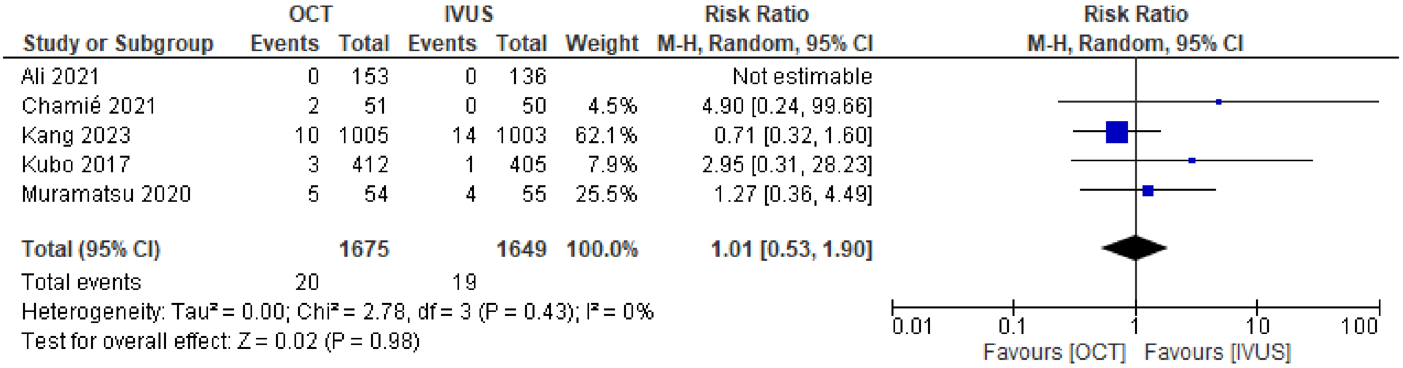

Supplement: Supplementary file 1 [file Datasheet1.pdf]
